# Novel biomarker SARIFA in colorectal cancer: highly prognostic, not genetically driven and histologic indicator of a distinct tumor biology

**DOI:** 10.1038/s41417-023-00695-y

**Published:** 2023-11-22

**Authors:** Nic G. Reitsam, Veselin Grozdanov, Chiara M. L. Löffler, Hannah S. Muti, Bianca Grosser, Jakob N. Kather, Bruno Märkl

**Affiliations:** 1 Pathology, Faculty of Medicine, University of Augsburg, Augsburg, Germany; 2 Bavarian Cancer Research Center (BZKF), Augsburg, Germany; 3 Department of Neurology, Ulm University, Ulm, Germany; 4 Else Kroener Fresenius Center for Digital Health, Technical University Dresden, Dresden, Germany; 5 Department of Medicine I, University Hospital Dresden, Dresden, Germany; 6 Department of Visceral, Thoracic and Vascular Surgery, University Hospital Carl Gustav Carus Dresden, Dresden, Germany; 7 Pathology & Data Analytics, Leeds Institute of Medical Research at St James’s, University of Leeds, Leeds, UK; 8 Medical Oncology, National Center for Tumor Diseases (NCT), University Hospital Heidelberg, Heidelberg, Germany

**Keywords:** Cancer, Biomarkers

## Abstract

SARIFA (Stroma AReactive Invasion Front Areas) has recently emerged as a promising histopathological biomarker for colon and gastric cancer. To elucidate the underlying tumor biology, we assessed SARIFA-status in tissue specimens from The-Cancer-Genome-Atlas (TCGA) cohorts COAD (colonic adenocarcinoma) and READ (rectal adenocarcinoma). For the final analysis, 207 CRC patients could be included, consisting of 69 SARIFA-positive and 138 SARIFA-negative cases. In this external validation cohort, H&E-based SARIFA-positivity was strongly correlated with unfavorable overall, disease-specific, and progression-free survival, partly outperforming conventional prognostic factors. SARIFA-positivity was not associated with known high-risk genetic profiles, such as *BRAF V600E* mutations or microsatellite-stable status. Transcriptionally, SARIFA-positive CRCs exhibited an overlap with CRC consensus molecular subtypes CMS1 and CMS4, along with distinct differential gene expression patterns, linked to lipid metabolism and increased stromal cell infiltration scores (SIIS). Gene-expression-based drug sensitivity prediction revealed a differential treatment response in SARIFA-positive CRCs. In conclusion, SARIFA represents the H&E-based counterpart of an aggressive tumor biology, demonstrating a partial overlap with CMS1/4 and also adding a further biological layer related to lipid metabolism. Our findings underscore SARIFA-status as an ideal biomarker for refined patient stratification and novel drug developments, particularly given its cost-effective assessment based on routinely available H&E slides.

## Background

With more than 1.8 million new cases every year, colorectal cancer (CRC) is the third most common cancer, contributing extensively to the global burden of disease [1]. Even though the 5-year overall survival (OS) rate is approximately 90% for early stages, this rate rapidly decreases for later stages [2].

In clinical practice, prognosis evaluation and treatment guidance in CRC patients is based on the American Joint Committee on Cancer/Union for International Cancer Control/Tumor Node Metastasis (AJCC/UICC/TNM) classification. Besides that, additional prognostic factors such as grade, tumor budding, *KRAS* and *BRAF* mutational status, and mismatch repair (MMR) or microsatellite status have been established. However, TNM staging as well as other routinely used biomarkers are still not fully sufficient to predict the survival of stage II and III patients as these are associated with extremely divergent patient outcomes. For example, stage IIIA patients do have a better prognosis than stage IIB/IIC patients, which is described as the *“survival paradox”* [3]. Considering that adjuvant therapy with fluoropyrimidine + oxaliplatin-based chemotherapy is recommended following surgical treatment for a really heterogeneous group of stage III as well as high-risk stage II patients [4], it is evident that there is still a lack of biomarkers sufficiently stratifying CRC patients, leading to under- or overtreatment of some patients.

Recently, gene-expression-based approaches such as consensus molecular subtyping (CMS) [5] or CINSARC signatures [6], that have impressively been shown to correlate with prognosis and partly even outperform conventional TNM staging, have been established. However, these RNA-sequencing-based methods are not easily applicable and time- as well as cost-intensive, and therefore have not found their way into the daily clinical routine yet.

To meet this urgent need for new robust and easy-to-implement biomarkers in CRC, we recently introduced Stroma AReactive Invasion Front Areas (SARIFA) as hematoxylin and eosin (H&E) based negative predictor in colon [7] as well as gastric cancer [8]. SARIFA, which is defined as the direct contact between tumor cells and adipocytes at the invasion front, shows a low interobserver variability and can be assessed fast and easily on routine H&E slides. Hence, there is no need for further testing (via e.g., immunohistochemistry or sequencing), and hence no delay in turnaround time and no additional costs (except for the pathologist’s effort).

Recently, our group provided the first evidence that SARIFA is associated with tumor-promoting tumor-adipocyte interaction [8] as well as deleterious immunologic alterations [9]. Upregulation of proteins associated with fatty acid metabolism such as *FABP4* and *CD36* in tumor cells at the invasive margin seems to be a key feature of SARIFAs [8], which is already known to contribute to the invasive and metastatic potential of colon cancer cells [10, 11], and could be a potential innovative therapeutic target in SARIFA-positive CRCs [12–14].

Besides numerous experimental studies highlighting the major role of adipocytes and lipids in cancer progression [15, 16], two independent deep-learning models have just recently identified tumor cell/adipocyte co-localization, as important. This is similar to what we define as SARIFA and has so far been an underappreciated morphological feature associated with a worse prognosis in CRC [17, 18].

Hence, it is reasonable that our H&E-based SARIFA classification represents an aggressive tumor biology, which is characterized by a distinct tumor-adipocyte interaction, potentially caused by immunologic dysregulation.

Therefore, this study aims to validate the prognostic relevance of SARIFA in CRC on The-Cancer-Genome-Atlas (TCGA) colonic (COAD) and rectal adenocarcinoma (READ) cohorts and to provide the first in-depth molecular characterization of SARIFA-positive CRCs in a well-characterized, publicly available external cohort [19].

## Methods and materials

### Ethics statement

The experiments in this study are in compliance with the Declaration of Helsinki and the International Ethical Guidelines for Biomedical Research Involving Human Subjects by the Council for International Organizations of Medical Sciences (CIOMS). The study has been carried out according to the “Transparent reporting of a multivariable prediction model for individual prognosis or diagnosis” (TRIPOD) statement [20]. The overall analysis in this study has been approved by the Ethics Board at the Medical Faculty of Technical University Dresden (BO-EK-444102022). The patient sample collection in each cohort was separately approved by the respective institutional ethics boards.

### Data acquisition

Whole slide images (WSI) were obtained from https://portal.gdc.cancer.gov/ for *n* = 627 colorectal (CRC) patients from TCGA cohorts COAD (colonic adenocarcinoma) and READ [19]. Patients were then histopathologically screened regarding their SARIFA-status by a pathologist with plenty of experience in assessing SARIFA-status (first author, NGR). In total, *n* = 215 could be classified into SARIFA-positive and SARIFA-negative. Molecular data are available at https://www.cbioportal.org/ for the TCGA PanCancerAtlas [21, 22]. Additional data on the datasets were partly retrieved and are available from Liu et al. [23], from Thorsson et al. [24] as well as from Malta et al. [25].

### Assessment of SARIFA-status

**Fig. 1:**
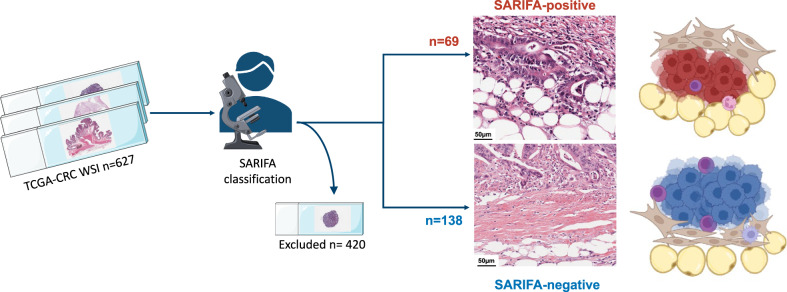
Study design and SARIFA definition. TCGA cohorts READ and COAD (TCGA-CRC) were screened for suitable cases. Overall, 420 cases were excluded from the final analysis. In most cases only superficial tumor parts and not the tumor-fat-interface, which is necessary for adequate SARIFA assessment, were depicted. Here, 207 cases could be reliably classified based on available WSIs and with sufficient clinical data available and were therefore used for further analysis. SARIFA is a solely H&E-based biomarker, which is defined by the direct contact between adipocytes and tumor cells at the invasion front (SARIFA-positive). If there is a desmoplastic reaction or inflammation in between tumor cells and adipocytes, cases were assessed as SARIFA-negative. COAD colonic adenocarcinoma, READ rectal adenocarcinoma, CRC colorectal cancer, H&E hematoxylin and eosin, SARIFA Stroma AReactive Invasion Front Areas, TCGA The Cancer Genome Atlas, WSI whole slide images. Created with BioRender.com and Smart Medical Art.

As the digital slides at https://portal.gdc.cancer.gov and http://www.cbioportal.org/ are consistently scalable and therefore also have been used for other morphologic characterization studies [26], SARIFA-status could be reliably assessed when diagnostic WSI with the complete intestinal wall and not only superficial tumor parts were available (*n* = 215). SARIFA-positivity was defined according to our previous studies in CRCs [7, 9] as the presence of an area within the tumor invasion front where at least a single tumor gland or group of ≥5 tumor cells are directly adjacent to adipocytes (SARIFA-positive, Fig. 1) without intervening stromal reaction or inflammatory infiltrate (SARIFA-negative, Fig. 1). If a single SARIFA was present, the case was classified as SARIFA-positive. All the cases were classified by NGR. Clinical and molecular data (TCGA PanCancerAtlas) of *n* = 207 classified cases were partly analyzed and could be retrieved from http://www.cbioportal.org/ [19, 21, 22].

### Statistical analysis and experimental design

Chi-squared tests were used for hypothesis testing of differences between relative frequencies. Continuous variables were compared using the Wilcoxon rank-sum test. Estimates of Kaplan–Meier survival rates were compared using log-rank tests. The median follow-up was calculated using the reverse Kaplan-Meier method [27]. Relative risks were estimated by hazard ratios (HRs), obtained by Cox proportional hazard models. For genomic alterations, mRNA and protein expression, methylation data, and microbiome signatures, *q*-values are reported to incorporate multiple testing corrections (using a false discovery rate detection approach). Cramér’s *V* was also reported for association between nominal variables. *p*-Values < 0.05 were considered statistically significant and are reported as follows: *p < 0.05, **p < 0.01, ***p < 0.001, ****p < 0.0001. Statistical analyses were performed within http://www.cbioportal.org/ and by using R (v4.2.2; R Foundation for Statistical Computing, Vienna, Austria) or SPSS for Windows, version 24 (IBM, Armonk, NY, USA). The study design is visualized in detail in Fig. 1.

### Gene and protein expression analysis

For gene expression analysis, the batch-normalized RNA-seq data generated with RSEM [28] from Illumina HiSeq_RNASeq_V2 *(‘rna_seq_v2_mrna’)* data was accessed from TCGA over *cBioPortal*. Gene expression data were available for 207/215 (96%) TCGA samples with known SARIFA-status. Missing values were replaced with zero counts. Among the samples, 11 samples could not be considered because of negative counts due to the batch correction. Differential expression analysis was performed with DESeq2 v1.36.0 [29] with counts rounded to an integer and Wald test without covariates or with sex included as a covariate in the linear model. CMS subtyping of samples was performed with CMScaller v2.0.1 [30] with the raw counts and with Entrez gene identifiers, 5000 permutations *(random seed 31415),* and an FDR (false discovery rate) threshold of 0.05. In both groups, 12–14% of the samples could not be confidently assigned a CMS subtype. Functional gene set analysis was performed with GO (Panther v16.0 with GO db as of 12/2021) [31], ShinyGo v0.77 [32], and GSEA (gene set enrichment analysis) v4.3.2 [33]. Gene and protein expression data were visualized with ggplot2 v3.4.2 [34] and networkD3 v0.4. Protein expression was accessed as processed RPPA (reverse phase protein assay) values from *cBioPortal* and visualized with ggplot2. Drug response predictions based on gene expression were established by deploying *oncoPredict* [35] with training data from the GDSC2 database [36]. The prediction model was run primarily with default settings (https://cran.r-project.org/web/packages/oncoPredict/oncoPredict.pdf).

### Availability of molecular and image data

The dataset(s) supporting the conclusions of this article are included within the article (and its additional files). Molecular and image data are publicly available at https://portal.gdc.cancer.gov/ and https://www.cbioportal.org/.

SARIFA-status of the cases can also be found in detail in Additional File 1.

## Results

### SARIFA is associated with poor outcomes in TCGA-CRC

In our classified TCGA-CRC cases (*n* = 207), overall 69 patients (33.3%) presented with SARIFA-positive CRC. While age at diagnosis, patient weight, and length of follow-up did not differ based on SARIFA-status, SARIFA-positivity in primary CRC tumor tissue was significantly associated with adverse features such as higher pT stage, lymph node and distant metastasis, higher AJCC stage, occurrences of deaths and new neoplasms post initial therapy (each *p*-value at least <0.01, chi-squared test). Regarding MSI (microsatellite instability), *BRAF* status, and TCGA subtypes based on driver mutations, no SARIFA-dependent differences were evident. Clinicopathological features of the cohort with regard to SARIFA-status are displayed in detail in Table 1.

**Table 1:** Clinicopathological Characteristics of TCGA cohorts COAD and READ with regards to SARIFA.

		All cases		SARIFA-positive		SARIFA-negative		*p*-Value
			in %		in %		in %	
		*n* = 207	100.0	*n* = 69	33.3	*n* = 138	66.7	
Median age (range), years		67 (31–90)		68 (35–90)		67 (31.5–90)		0.844
Median patient weight (range), kg [*n* = 178 of all, 29 NA]		77.5 (42–129)		77.2 (45.9–124)		77.5 (42–129)		0.917
Median follow-up (95% CI) months		26.4 (19.9–32.9)		26.0 (15.8–36.2)		27.6 (18.8–36.3)		0.580
Sex								0.694
	Female	103	49.76	33	47.8	70	50.7	
	Male	104	50.24	36	52.2	68	49.3	
Location								0.898
	Colon	170	82.1	57	82.6	113	81.9	
	Rectum	37	17.9	12	17.4	25	18.1	
T Status								**0.009**
	≤pT2	31	15.0	4	5.8	27	19.6	
	≥pT3	176	85.0	65	94.2	111	80.4	
N Status								**0.002**
	negative	109	52.7	26	37.7	83	60.1	
	positive	97	46.9	43	62.3	54	39.1	
	NA	1	0.5	0	0.0	1	0.7	
Distant metastasis								**<0.001**
	no	141	67.6	43	62.3	98	71.0	
	yes	30	14.5	19	27.5	11	8.0	
	NA	36	17.4	7	10.1	29	21.0	
AJCC								**<0.001**
	I/II	104	50.2	23	33.3	81	58.7	
	III/IV	98	47.3	44	63.8	54	39.1	
	NA	5	2.4	2	2.9	3	2.2	
Microsatellite status								
MSI mantis score > 0.4								0.687
	MSS	174	84.1	57	82.6	117	84.8	
	MSI	33	15.9	12	17.4	21	15.2	
MSI sensor > 3.5								0.770
	MSS	178	86.0	60	87.0	118	85.5	
	MSI	29	14.0	9	13.0	20	14.5	
*BRAF* Mutational Status								0.777
	wildtype	181	87.4	60	87.0	121	87.7	
	mutant	26	12.6	9	13.0	17	12.3	

*p*-Values from Pearson’s chi-squared test are shown for the difference between SARIFA-positive and -negative CRC patients.

*NA* not available, *AJCC* American Joint Committee on Cancer, *MSS* microsatellite stable, *MSI* microsatellite instable, *T* depth of tumor invasion, *N* lymph node status, *CI* confidence interval.

**Fig. 2:**
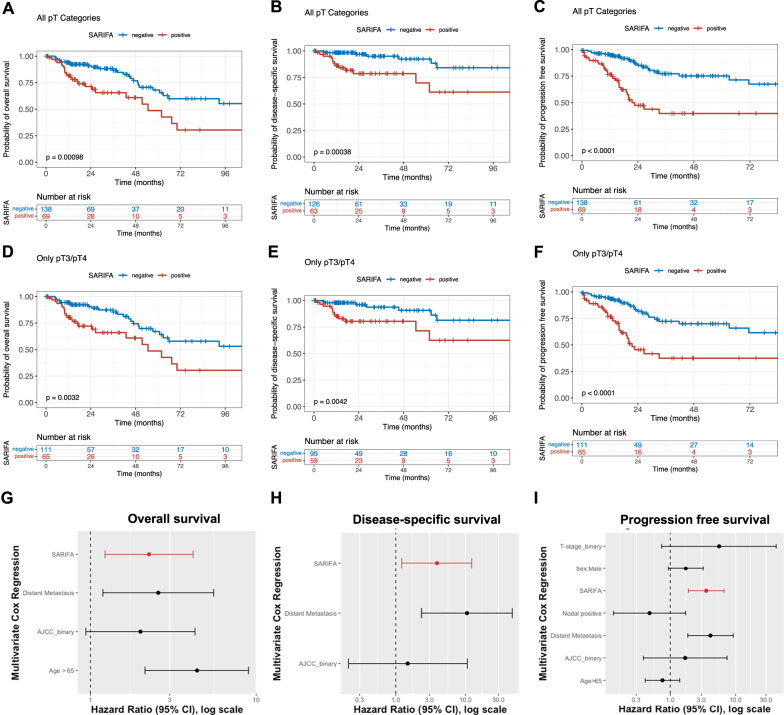
Kaplan–Meier curves of colorectal cancer patients in TCGA based on SARIFA-status. Survival rates differ significantly depending on SARIFA-status. **A**–**C** All T stages included. **D**–**F** Only T3 and T4 (T4a as well as T4b) tumors were included. **A**, **D** Overall survival. **B**, **E**: Disease-specific survival. **C**, **F** Progression-free survival. **G**–**I** Forest plots of multivariate Cox regression analysis adjusted for all parameters that were statistically significant in univariate analysis. SARIFA-negative, blue; SARIFA-positive, red. SARIFA Stroma AReactive Invasion Front Areas.

Next, we studied survival endpoints using Kaplan–Meier analysis (Fig. 2A–F). Here, SARIFA-positive CRC patients were characterized by a significantly decreased OS, progression-free survival (PFS), and disease-specific survival (DSS) throughout all T-stages and also when only considering locally advanced T3/T4 CRCs (each *p*-value at least **<0.01, log-rank test). In particular, Kaplan–Meier curves regarding PFS separated impressively (median months PFS: SARIFA-positive: 22.62 [19.04–NA], SARIFA-negative: not reached).

To assess the value of SARIFA-status in comparison to other prognostically relevant factors, we performed Cox regression analysis (Additional File 2) including parameters such as age (>65 years), gender, pT, pN, and M status, AJCC stage and MSI scores (Mantis & Sensor). Upon univariate analysis regarding OS, higher age, presence of distant metastasis, higher AJCC stage, and SARIFA-positivity were significantly associated with worse outcomes, with distant metastasis and SARIFA-positivity having the highest HRs of 3.12 and 2.43, respectively (HR: distant metastasis 95% CI: 1.64–5.97, ***<0.001; SARIFA 95% CI: 1.411–4.212). Regarding DSS and PFS, SARIFA-positivity was also significantly associated with adverse outcomes (DSS, HR: 4.58 95% CI: 1.82–11.50, *p* = 0.001; PFS, HR: 3.66 95% CI: 2.13–6.30, ***p < 0.001). Consecutively, we performed multivariate regression analysis, including only the parameters that were statistically significant in univariate analysis (Fig. 2G–I). Here, SARIFA-positivity remained highly prognostic with regards to all three endpoints (OS, HR: 2.5 95% CI: 1.22–4.14, **p = 0.009; DSS, HR 3.87 95% CI: 1.22–12.31 *p = 0.022; PFS 3.56 95% CI: 1.89–6.69, ***p < 0.001). With SARIFA-status showing consistently higher HRs (however, with partly overlapping CIs) than conventional biomarkers, we confirm SARIFA as possibly superior, solely H&E-based biomarker that potentially outperforms prognostic biomarkers that are currently used to guide treatment decisions in CRC patients.

### SARIFA is not associated with distinct genetic alterations

**Fig. 3:**
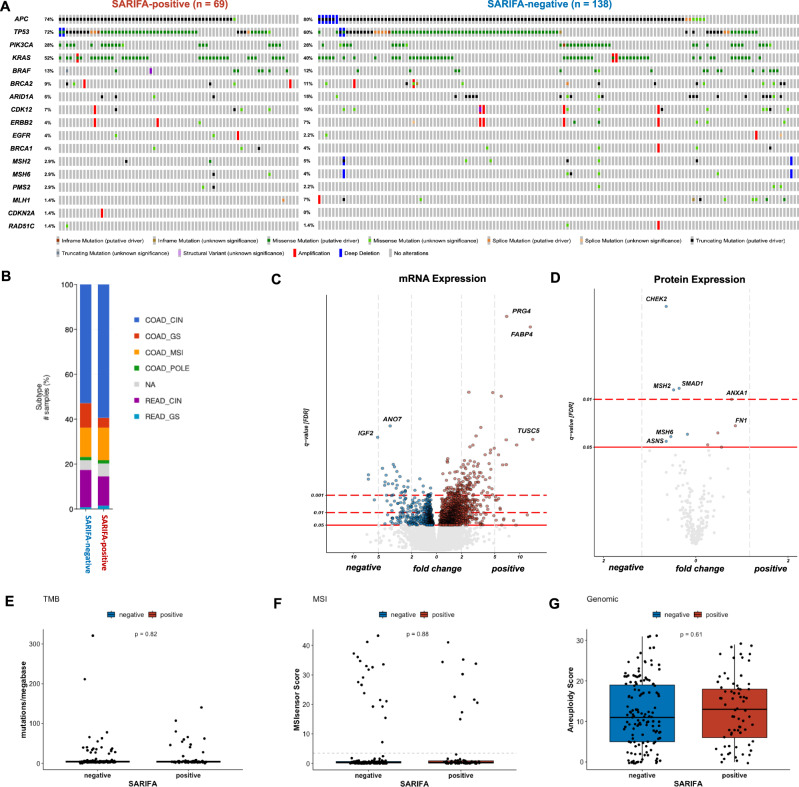
Comprehensive molecular characterization with regards to SARIFA-status. **A** Oncoprint showing the mutational profile of SARIFA-positive and SARIFA-negative CRCs. **B** Molecular subtype distribution. **C** Differential gene expression analysis. **D** Protein expression analysis. **E** Tumor mutational burden. **F** MSIsensor scores (with a cut-off of 3.5 as a dashed line). **G** Aneuploidy score. COAD colonic adenocarcinoma, CRC colorectal cancer, CIN chromosomal instable, GS genomic stable, POLE DNA polymerase epsilon, SARIFA Stroma AReactive Invasion Front Areas, TMB tumor mutational burden, READ rectal adenocarcinoma, MSI microsatellite instability.

As survival outcomes differ dramatically between SARIFA-positive and SARIFA-negative CRCs, we aimed to explore if genetic alterations drive these differences and took the in-depth characterization published by Liu et al. in their landmark study on molecular characteristics of gastrointestinal adenocarcinomas into account [23]. In line with our previous findings based on immunohistochemistry-based TCGA molecular subtyping in GC [8] or on small next-generation-sequencing (NGS) panel approaches in CRC [9], we could not find any significant SARIFA-dependent differences on the genomic level (no significant sample-level enrichments, no significant differences in tumor mutational burden, fraction genome altered or aneuploidy score; no significant differences in indel mutation density, SNV (single nucleotide variant) mutation density and total mutation density as well as the fraction of genome with subclonal SCNAs (somatic copy number alterations) and duplicated alleles, all *p* > 0.05). Genomic alterations regarding the most relevant genes in CRC are paradigmatically visualized as Oncoprint in Fig. 3. In particular, SARIFA-positivity was not associated with known high-risk features such as *BRAF V600E* mutations (SARIFA-positive 10.4% vs. 8.0% SARIFA-negative, *p* = 0.27) or MSS (MSI Sensor score as well as MSI Mantis score, *p* > 0.05; MSI, microsatellite instable; MSS microsatellite stable). Regarding molecular subtypes (CIN, GS, MSI, and POLE) and hypermethylation category (CIMP-H, CIMP-L, and non-CIMP; CIMP: CpG island methylator phenotype), also no significant differences between SARIFA-positive and SARIFA-negative CRCs could be observed (molecular subtypes: *p* = 0.650, hypermethylation category: *p* = 0.441).

Analyzing the data within *cBioPortal* [21, 22], we could not observe any differences regarding DNA methylation data, and only one bacteria species (*Sutterella ssp*.) was observed, which seems upregulated in SARIFA-positive cases (****p = 6.0009e−6, *q* = 8.448e−3).

### SARIFA displays a characteristic gene expression signature and differential protein expression

**Fig. 4:**
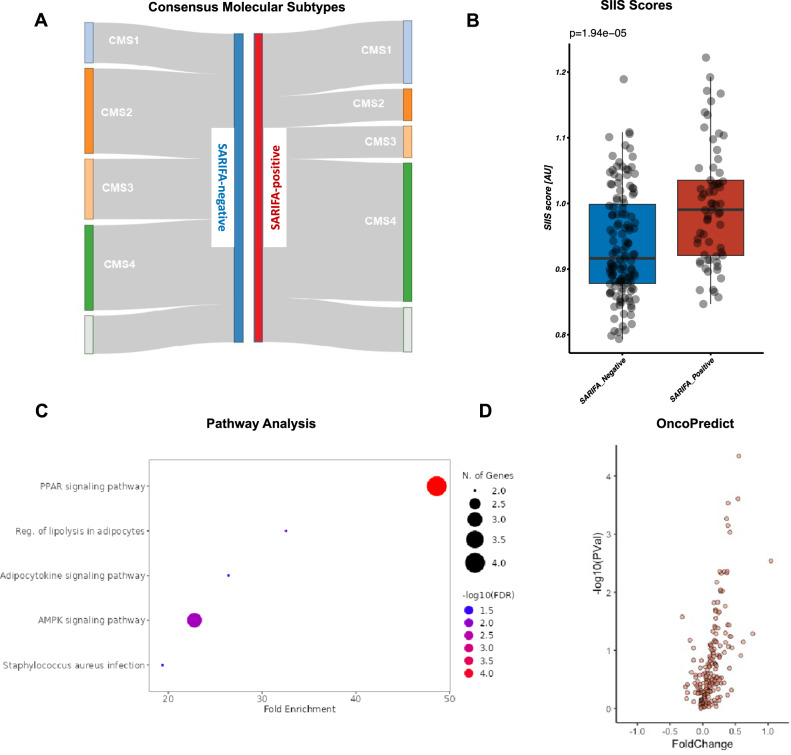
SARIFA-positive CRCs show a distinct gene expression profile. **A** Enrichment of CMS1 (MSI Immune) and especially CMS4 (mesenchymal) cases within SARIFA-positive CRCs. **B** Higher SIIS scores of SARIFA-positive CRCs. **C** Pathway analysis considering 27 genes that were significantly up- or downregulated more than 1.5-fold. **D** Differential drug sensitivity of SARIFA-positive CRCs based on *oncoPredict* (see also Additional File 5). CRC colorectal cancer, SARIFA Stroma AReactive Invasion Front Areas, CMS consensus molecular subtype, MSI microsatellite instability, SIIS stromal cell infiltration score.

In contrast to genetic alterations, SARIFA-status in CRCs was associated with distinct changes in gene expression on the mRNA and protein levels. Differential gene expression analysis with the transcriptome profiles of the 196 CRC samples with available transcriptomic data and SARIFA-status revealed a broad dysregulation of gene expression (1896 genes/~9.6% with *q* < 0.05 and no LFC threshold; 731 genes/~3% with *q* < 0.01 and no LFC threshold; LFC: log fold change), with the major proportion of differentially expressed genes (approximately two-thirds) up-regulated in SARIFA-positive cases (Fig. 3C). Differential gene expression was similar when adjusting for sex (803 genes, ~4.1% with *q* < 0.05 and no LFC threshold, sex information available in 189 cases), while we did not find significant differential gene expression in a random sample permutation control with balanced SARIFA-negative and SARIFA-positive cases (33 genes, ~0.1% with *q* < 0.05 and no LFC threshold, mean of the results of three random permutations with equal SARIFA proportions and similar sex proportion as in the SARIFA analysis). Detailed results of differential gene expression analysis can be found in the supplementary (Additional File 3). Gene ontology enrichment analysis revealed enrichment of extracellular matrix, proteoglycans, and signaling pathways (Additional File 4). 27 genes were significantly up or downregulated more than 1.5-fold (22 up/5 down, Wald’s test against a null hypothesis of 0.585 LFC), among which were *FABP4 and CD36*, which we previously identified as differentially expressed at SARIFAs in gastric cancer [8]. These 27 genes showed significantly enriched molecular interaction (PPI enrichment *p*-value: 3.23e−11, STRING db), expression in adipocytes and adipose tissue (TISSUES db), association with the extracellular space (Gene ontology and COMPARTMENTS db) and with the PPAR/AMPK signaling and adipocyte signaling pathways (GO, KEGG and WikiPathways db) (Fig. 4C), suggesting dysregulation of a functional network in SARIFA-positive CRCs. Interestingly, differential protein abundance analysis with reverse phase protein assay (RPPA) data demonstrated several differentially abundant proteins, which are also associated with the extracellular matrix (Fig. 3D).

Next, we investigated different gene-expression-based signatures and found several specific characteristics of SARIFA-positive CRCs. SARIFA-positive cases were associated with lower stemness (*p = 0.04); although there was no difference for DNA-methylation-based stemness (*p* = 0.71). Furthermore, SARIFA-positive CRCs were associated with higher stromal cell infiltration intensity (SIIS) scores [37] (Fig. 4B, ***p < 0.01, Mann–Whitney *U*-test). We also investigated if SARIFA-status is associated with distinct consensus molecular subtypes (CMS) [5] based on their RNA-expression profile. CMS profiles could be confidently assigned to 180/207 samples (87%). The distribution of the CMS profiles in SARIFA-positive cases differed significantly from SARIFA-negative cases (**p < 0.01, hypothesis test of equal population proportions, Cramér’s *V* φ_c_ = 0.29), with the relative proportion of CMS4 and CMS1 increased in SARIFA-positive cases (CMS4: 53% vs. 31%; CMS1: 24% vs. 15%) and CMS2 and CMS3 decreased in SARIFA-positive cases (CMS2: 12% vs. 31%; CMS3: 12% vs. 22%) (Fig. 4A). Thus, SARIFA-positivity was significantly associated with CMS1 and CMS4 molecular subtypes, but did not strongly overlap with the CMS subtyping of colorectal tumor samples.

Besides showing enrichment of CMS1 (immune), SARIFA-positive CRCs also displayed a significantly increased expression of *CD274* (*PD-L1*; LFC 0.91, ****p < 0.0001, *q*-value = 0.0032). Both findings could be relevant for immunotherapeutic approaches, and strengthen our previous finding of an altered immune response in SARIFA-positive CRCs [9].

Furthermore, as it is well-established that hypoxic tumors such as SARIFA-positive tumors are associated with high-risk features and poor outcomes [38], we investigated the relationship between three different hypoxia scores and did not find any SARIFA-dependent changes (Buffa hypoxia score: *p* = 0.799, Ragnum hypoxia score: *p* = 0.800, Winter hypoxia score: *p* = 0.267, Wilcoxon rank sum test).

### SARIFA-based gene expression pattern predicts differential therapy response

Based on the observed gene expression profiles, we further analyzed if differential gene expression leads to differences in predicted treatment responses. Therefore, we deployed *oncoPredict*, which is a computational tool to derive drug responses based on cell line screening data [35]. Here, indeed SARIFA-positive CRCs displayed a differential drug sensitivity (Fig. 4D as well as additional file 5). Among the 198 analyzed compounds, we could identify four drugs, which are currently used in the treatment of CRC patients, in the primary and/or in the metastatic setting (Oxaliplatin_1089, 5-Fluorouracil_1073 [5-FU], Irinotecan_1088, Lapatinib_1558). Interestingly, SARIFA-positive CRCs are predicted to be more resistant to Oxaliplatin with higher predicted IC50 values (fold change 1.045, *p* = 0.0029, *q* = 0.078). Whereas for 5-FU (fold change 0.44, *p* = 0.48, *q* = 0.14), Irinotecan (fold change 0.22, *p* = 0.29, *q* = 0.60), and Lapatinib (fold change 0.20, *p* = 0.83, *q* = 0.91) no significant differences could be observed. Moreover, SARIFA-positive CRCs seem, in line with their partial overlap with CMS4, more sensitive to Dasatinib, which is an FDA (U.S. Food and Drug Administration) approved tyrosine kinase inhibitor against CMS4-related kinases [39] and already in clinical use for chronic myeloid leukemia (Dasatinib_1079, fold change −0.31, *p* = 0.027, *q* = 0.22). At least a similar trend could be observed for further compounds, namely JQ1_2172 (fold change −0.19, *p* = 0.067, q = 0.34) and XAV939 (fold change −0.14, p = 0.092, q = 0.36). JQ1 has been described as an active drug in CRC cell lines and patient-derived xenografts [40], whereas XAV939 is supposed to function via inhibition of *Wnt/beta-catenin* signaling, which plays a central role in CRC [41].

## Discussion

Adequate patient stratification in CRC in routine diagnostic pathology, especially in TNM stages II/III [3], still remains challenging. To come up with a solution for this pressing clinical need, we established SARIFA-status as a solely H&E-based biomarker [7, 8], that could be fast and easily implemented in routine pathologic workflow straight away. Compared to tumor budding [42, 43], which is a histopathologic biomarker already in clinical use, SARIFA-status is characterized by a low interobserver variability without a need for further immunohistochemical stains or assays [7, 8]. To further characterize the prognostic relevance as well as the molecular background of SARIFA, we comprehensively investigated SARIFA as a biomarker in the openly available TCGA cohorts COAD and READ. Besides further insights into tumor biology with regards to SARIFA-status, this approach has the advantage of making our SARIFA assessment publicly available and thereby not only providing a training resource for pathologists but also serving as a starting point for further research efforts.

By deploying TCGA-CRC as the first publicly available, external validation cohort, we could again prove the association of SARIFA-positivity with known conventional high-risk features such as higher pT categories and positive lymph nodes. Furthermore, SARIFA-positivity was strongly associated with poor outcomes with regard to different endpoints, namely OS, PFS, and DSS, even within locally advanced (pT3/pT4) CRCs. SARIFA-status remained one of the strongest independent predictors with regard to all investigated endpoints upon multivariate analysis. In line with our findings, other groups just recently provided further evidence that adipocytes close to tumor cells are a morphological feature that is associated with a poor prognosis in CRC [18, 44]. Even though novel approaches with comparable performance to better stratify CRC patients based on gene-expression profiling or deep learning algorithms have recently been published [6, 18], SARIFA assessment does not rely on challenging assays or computing power but solely on H&E histopathology.

Based on extensive molecular profiling, that has been done within TCGA and further related studies, we could now prove that SARIFA and its associated poor prognosis is likely not driven by genetic changes as SARIFA-positivity was not associated with any harmful molecular changes such as deleterious *BRAF V600E* mutations [45] or MSS status [46], which are known to convey a poor prognosis. As our previous understanding of genetic alterations with regards to SARIFA-status in CRC was based on very limited sample numbers and only panel-based NGS sequencing [9], our current study confirms that SARIFA-positivity is not a reflection of harmful genetic alterations.

Furthermore, this is the first study that could prove that SARIFA-positive CRCs have a similar upregulation of fatty acid metabolism, just as observed in SARIFA-positive GCs. Strikingly, whereas we initially identified an upregulation of *FABP4* and *CD36*, both closely related to lipid metabolism, specifically at SARIFAs in GC [8], we now provide the first evidence that these genes are also upregulated in RNA bulk data. Even though it seems like a limitation, that only bulk data is available for TCGA-CRC, and bulk data is unlikely to reflect SARIFA as a spatially restricted process at the invasion front, our results show that bulk RNA-seq reflects robust gene expression changes associated with SARIFA not only at the invasion front but the entire tumor as well as the tumor microenvironment.

By linking our gene expression profiles of SARIFA-negative and SARIFA-positive CRCs to the established CMS subtypes [5] for the very first time, we identified an enrichment of CMS1 (immune) and especially CMS4 (mesenchymal) CRCs within SARIFA-positive cases. Moreover, there was a pronounced upregulation of genes associated with extracellular matrix organization such as *Proteoglycan 4*, and higher SIIS scores in SARIFA-positive CRCs, underlining their more mesenchymal phenotype. Previous studies, partly based on deep learning algorithms, could already detect a distinct genotype-phenotype correlation between histomorphologic features and CMS subtypes, such as the absence of mucin in CMS2 or desmoplastic reaction and high-grade budding in CMS4 CRCs [47–49]. Therefore, SARIFA-status based on H&E histopathology could serve as an indicator for CMS subtyping without the need for further cost-intensive RNA-based assays. As the enrichment of SARIFA-positive CRCs within CMS4 cases indicates, SARIFA-positive CRCs display a more mesenchymal, stroma-associated gene expression profile. Consequently, SARIFA-positive CRCs show significantly higher SIIS than SARIFA-negative CRCs. Higher stroma cell infiltration (higher SIIS) has already been proven as a high-risk feature in CRC and conveys an intrinsic drug resistance and therefore is associated with reduced efficacy of adjuvant chemotherapy [37]. Interestingly, SARIFA-negative CRCs showed an upregulation of *IGF2* (insulin-like growth factor 2), which has been recently described by Isella et al. as characteristic of CRC intrinsic subtype (CRIS) D [50]. In line with our findings in terms of prognosis, CMS4 CRCs, which have an overlap with SARIFA-positive CRCs, show the poorest prognosis [5], whereas CRIS-D CRCs, which show similarities to SARIFA-negative CRCs, seem to have the best outcomes [50].

Beyond this, SARIFA-positive CRCs were, as mentioned, characterized by an upregulation of genes associated with lipid metabolism, namely *FABP4* and *CD36*, which are known to play an important role in CRC as [10, 14, 51] well as general cancer progression [12, 52, 53], and hence could serve as novel therapeutic targets in SARIFA-positive CRCs [54, 55].

On the protein level, SARIFA-positive CRCs exhibited pronounced upregulation of Fibronectin and Annexin A, hinting on the one hand again at the key role of extracellular matrix organization with regards to SARIFA-status [56], and on the other hand on immunomodulatory changes within SARIFA-positive CRCs [57], which supports our previous findings of an altered immune response in SARIFA-positive CRC patients [9].

Finally, we investigated the predicted differential treatment response based on gene expression signatures of SARIFA-positive CRCs. Here, we observed a differential drug sensitivity. SARIFA-positive CRCs are predicted to be more sensitive to tyrosine kinase inhibitor (TKI) Dasatinib, which is in line with the findings that the use of TKIs in mesenchymal CMS4, which partly overlaps with SARIFA-positivity, can be beneficial [39]. Gene-expression-based drug sensitivity testing also suggested that SARIFA-positive CRCs are more resistant to Oxaliplatin treatment, which is part of most CRC chemotherapy regimens [4, 58], and therefore is of high clinical relevance. This finding is also in line with the higher SIIS observed in SARIFA-positive CRCs as higher SIIS indicates less benefit from adjuvant chemotherapy, as published previously [37]. Consistent with higher SIIS and the overlap with CMS4, a very recent study by Hu et al. could show that SARIFA-positivity is associated with non-mature desmoplastic reaction (with histologically visible keloid-like collagen [intermediate/middle desmoplastic reaction] or myxoid stroma [immature desmoplastic reaction]) [59]. Interestingly, the authors could also show that non-mature desmoplastic reaction as an H&E-based biomarker can potentially guide treatment decisions [59]. Beyond validating our findings that SARIFA-positivity is closely linked to changes in the extracellular matrix organization, these results highlight the important role of histologic biomarkers reflecting changes in the tumor stroma to predict treatment response.

To conclude, SARIFA-status is an independent and adverse prognostic histopathologic biomarker that does not only show some overlap with CMS1/CMS4 subtypes and high SIIS scores but also seems to possess a strong association with lipid metabolism. Therefore, we firmly believe H&E-based SARIFA-status is the equivalent of underlying aggressive tumor biology with its own transcriptional identity, which does not rely on genomic changes. We provide here the first external validation of SARIFA-status as a novel biomarker in CRC, which is based on an openly available data set and can therefore be used as a training resource for pathologists and researchers globally. SARIFA-status could be implemented easily and without further costs in routine diagnostic pathology and should be further validated in prospective trials as our current study also provides evidence that SARIFA-positive CRCs are characterized by a differential drug sensitivity.

### Supplementary information

Additional File 1

Additional Table 2

Additional File 3

Additional File 4

Additional File 5

## Data Availability

The dataset(s) supporting the conclusions of this article are included within the article (and its additional files). Moreover, molecular and image data are publicly available at https://portal.gdc.cancer.gov/ and https://www.cbioportal.org/. The Cancer-Genome-Atlas (TCGA) cohorts COAD (colonic adenocarcinoma) and READ (rectal adenocarcinoma) together with the corresponding WSI to each case are publicly available [19]. SARIFA-status of the cases can also be found in detail in Additional File 1. Molecular data is available at https://www.cbioportal.org/ for the TCGA PanCancerAtlas [21, 22]. Additional data on the datasets are available from Liu et al. [23], from Thorsson et al. [24] as well as from Malta et al. [25].
