# Supplementary material for: Novel biomarker SARIFA in colorectal cancer: highly prognostic, not genetically driven and histologic indicator of a distinct tumor biology
Source: Cancer Gene Ther. 2023 Nov 22;31(2):207–16. doi: 10.1038/s41417-023-00695-y (PMC10874891; doi:10.1038/s41417-023-00695-y)
Supplement: Supplementary file 4 — Additional File 4 [file 41417_2023_695_MOESM4_ESM.docx]

# Supplementary Figure


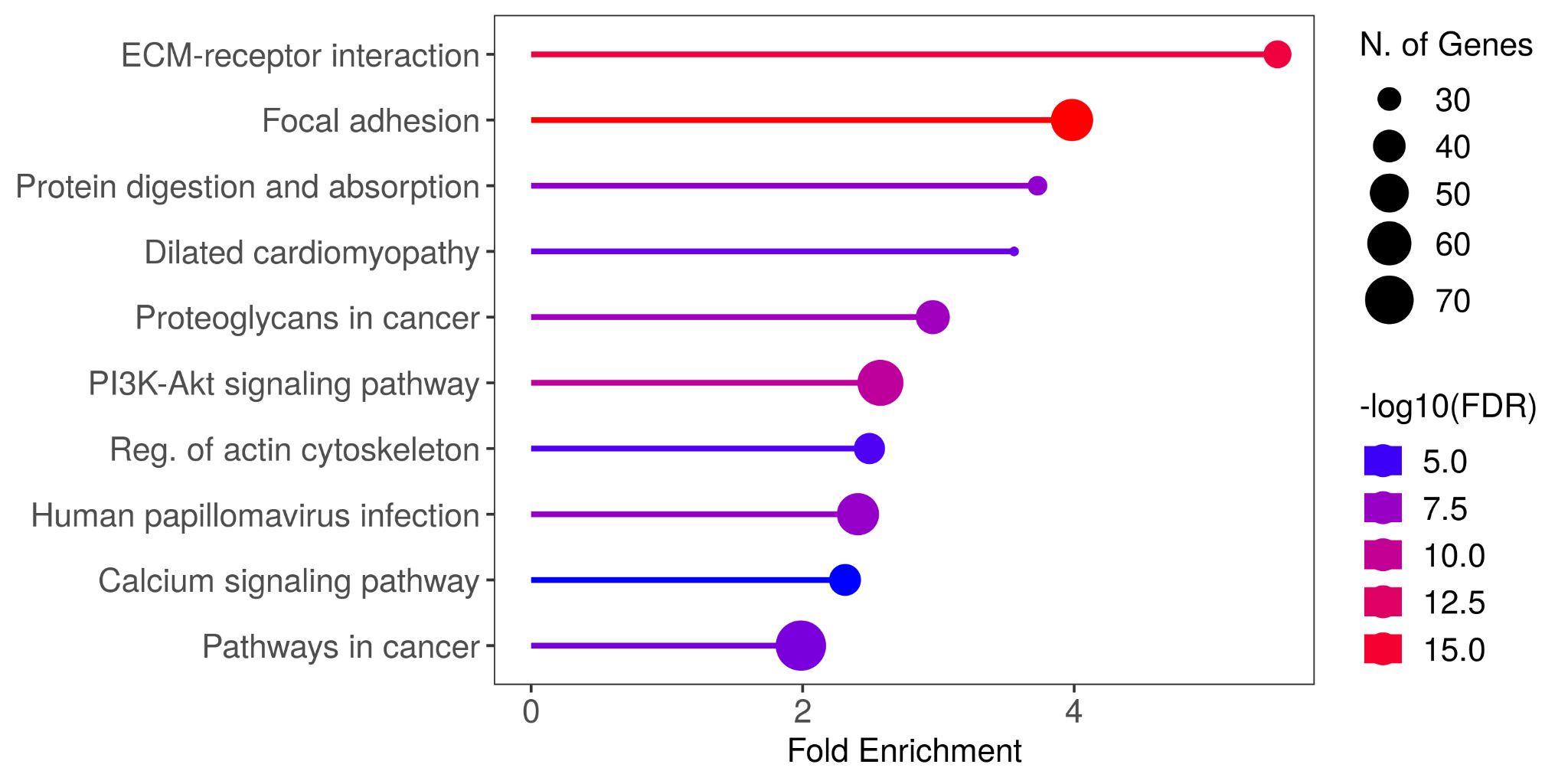


**Additional File 4. Gene ontology enrichment analysis revealed enrichment of extracellular matrix, proteoglycans and signaling pathways.** SARIFA-positive CRCs display a differential gene expression pattern, which leads to an enrichment of several biologically relevant pathways such as ECM-receptor interaction.

CRC: colorectal cancer, SARIFA: Stroma AReactive Invasion Front Area, ECM: extracellular matrix organization.
